# Long Term Hippocampal and Cortical Changes Induced by Maternal Deprivation and Neonatal Leptin Treatment in Male and Female Rats

**DOI:** 10.1371/journal.pone.0137283

**Published:** 2015-09-18

**Authors:** Virginia Mela, Francisca Díaz, Erika Borcel, Jesús Argente, Julie A. Chowen, Maria-Paz Viveros

**Affiliations:** 1 Department of Physiology (Anim Physiol II), Faculty of Biology, Complutense University Madrid, Madrid, Spain; 2 Department of Endocrinology, Hospital Infantil Universitario Niño Jesús, Instituto de Investigación la Princesa & CIBEROBN Instituto Carlos III, Madrid, Spain; 3 Brain Mind Institute and School of Life Sciences, Ecole Polytechnique Fédérale de Lausanne (EPFL), Lausanne, Switzerland; University of Amsterdam, NETHERLANDS

## Abstract

Maternal deprivation (MD) during neonatal life has diverse long-term behavioral effects and alters the development of the hippocampus and frontal cortex, with several of these effects being sexually dimorphic. MD animals show a marked reduction in their circulating leptin levels, not only during the MD period, but also several days later (PND 13). A neonatal leptin surge occurs in rodents (beginning around PND 5 and peaking between PND 9 and 10) that has an important neurotrophic role. We hypothesized that the deficient neonatal leptin signaling of MD rats could be involved in the altered development of their hippocampus and frontal cortex. Accordingly, a neonatal leptin treatment in MD rats would at least in part counteract their neurobehavioural alterations. MD was carried out in Wistar rats for 24 h on PND 9. Male and female MD and control rats were treated from PND 9 to 13 with rat leptin (3 mg/kg/day sc) or vehicle. In adulthood, the animals were submitted to the open field, novel object memory test and the elevated plus maze test of anxiety. Neuronal and glial population markers, components of the glutamatergic and cannabinoid systems and diverse synaptic plasticity markers were evaluated by PCR and/or western blotting. Main results include: 1) In some of the parameters analyzed, neonatal leptin treatment reversed the effects of MD (*eg*., mRNA expression of hippocampal IGF1 and protein expression of GFAP and vimentin) partially confirming our hypothesis; 2) The neonatal leptin treatment, *per se*, exerted a number of behavioral (increased anxiety) and neural effects (*eg*., expression of the following proteins: NG2, NeuN, PSD95, NCAM, synaptophysin). Most of these effects were sex dependent. An adequate neonatal leptin level (avoiding excess and deficiency) appears to be necessary for its correct neuro-programing effect.

## Introduction

Increasing evidence gives support to the fact that early-life stress induces permanent alterations in neurodevelopment [1] that may increase the risk of psychopathology at adulthood [2–4]. Manipulation of mother infant interactions has been extensively used as animal models of early-life stress [5]. Notably, among these models, maternal deprivation (MD), 24 h at postnatal day (PND) 9, has been used as an animal model of early-life stress highly suitable for the investigation of the developmental origin of certain psychiatric disorders such as schizophrenia and depression [6, 7]. MD has been reported to provoke behavioral abnormalities that resemble psychotic-like symptoms such a disruption in the pre-pulse inhibition (PPI) response [6, 8], neuroendocrine alterations related to stress reactivity [9–11], as well as cognitive impairments in adult animals [12]. Studies have also described deviant behaviors in maternally deprived adolescent animals, i.e. depressive-like responses [13] and increased impulsivity [14], as well as cognitive impairment [15]. In addition to these long-term behavioral outcomes, several short and long term brain alterations have been found, affecting neurons and glial cells, growth factors and diverse synaptic plasticity proteins in the hippocampus, cerebral cortex and hypothalamus of MD rats [16]. It is unclear how MD can modify these parameters since there are numerous stressors included in the MD protocol such as lack of maternal care and nutrients during the entire deprivation period, dehydration, and also a decrease of body temperature in MD pups [17–19]. One of the most striking effects of MD is the marked reduction in circulating leptin levels, not only during the maternal separation period (PND 9–10), but also several days after (PND 13) and in the adulthood [16].

Leptin, an adipokine produced mainly by adipocytes, is a pleiotropic hormone involved in many physiological processes such as food intake and energy balance, immune system functions, reproduction, reward, stress and neurodevelopment [20–22]. Regarding this latter role, there is a postnatal leptin surge in rodents, beginning around postnatal day (PND) 5 in males and peaking between PND9 and 10 [23], that has been implicated in hypothalamic development by modifying neuronal outgrowth and synaptic connectivity, as well as neurogenesis and neuronal and glial survival [24]. Though most of the work on the physiological role of this neonatal leptin signaling has focused on hypothalamic development [24–27], it is likely that it has also a crucial role on the development of other brain areas such as the hippocampus and the frontal cortex. There is ample evidence that supports this hypothesis. The leptin receptor (LepR) is expressed not only in the hypothalamus, but also in many other brain regions including the cortex, amygdala, cerebellum, brain stem, substantia nigra, hippocampal CA1, CA3 areas and dentate gyrus (DG) in rodents and humans [28–30]. Leptin receptors are present on the soma and proximal dendrites at presynaptic terminals of hippocampal neurons, as well as on hippocampal astrocytes [31, 32], and there is good evidence indicating a neurothropic and morphothropic role of leptin in this brain region [29, 30, 33–35]. The cortex also expresses high levels of LepRb mRNA during development [36, 37] and leptin deficiency results in a reduction in the number of cortical neurons born during embryonic life [38]. Leptin also appears to influence axonal growth in the developing cortex since it causes a marked increase in expansion of the axonal growth cone of primary cultures of embryonic cortical neurons. Leptin is also involved in the development of non-neuronal cells in the cortex and may influence the development of oligodendroglial cells [39].

As circulating leptin levels are dramatically reduced during MD, we hypothesized that exogenous leptin treatment would normalize at least some of the long-term effects on the hippocampus and cerebral cortex, as well as some of the behavioral alterations seen in adult MD rats. The aim of this study was to determine if a daily leptin treatment, between PND9 to PND13, could protect from deleterious effects of MD or palliate in any way some of changes induced by the MD protocol. We looked at behavioral and molecular parameters that we had previously analyzed and that were modified by MD [40]. Notably, important sex differences have been consistently described in relation to both the behavioral and neurobiological consequences of MD. Indeed, sex differences have been reported from early neonatal stages (PND 13) to adulthood [16]; thus, we have included both males and females in this study.

## Materials and Methods

### Animals

Adult Wistar rats were purchased from Harlan Interfauna Ibérica S.A. (Barcelona, Spain) and allowed to acclimate for 2 weeks before mating. One male was placed in a cage with two females for 10 days. On the day of birth (PND0), litters were culled to eight pups per dam (four males and four females). No cross-fostering was employed. In all experimental groups three different litters were used to reduce the litter effect, with a total of 12 rats in each experimental group. Rats were maintained at a constant temperature (22 ± 1°C) and humidity (50 ± 2%) in a reversed 12-h light-dark cycle (red light on at 08:00 and white light on at 20:00) and given free access to rat chow (commercial diet for rodents 2918; Harlan Laboratories, Madison, WI, USA) and water.

Experiments were approved by the local Animal Ethics Committee (Animal Research Committe of the Complutense University of Madrid), and were designated and performed in compliance with the Royal Decree 1201/2005, October 21, 2005 (Boletín Oficial del Estado,BOE n° 252) about protection of experimental animals, and the European Communities Council Directive of 24 November 1986 (86/609/EEC). The experiments were approved by the Animal Research Commitee of the Complutense University, reference number of the Protocol approval document: CA UCM 5 2012. Animals were sacrificed by decapitation

### Maternal deprivation

**Fig 1:**
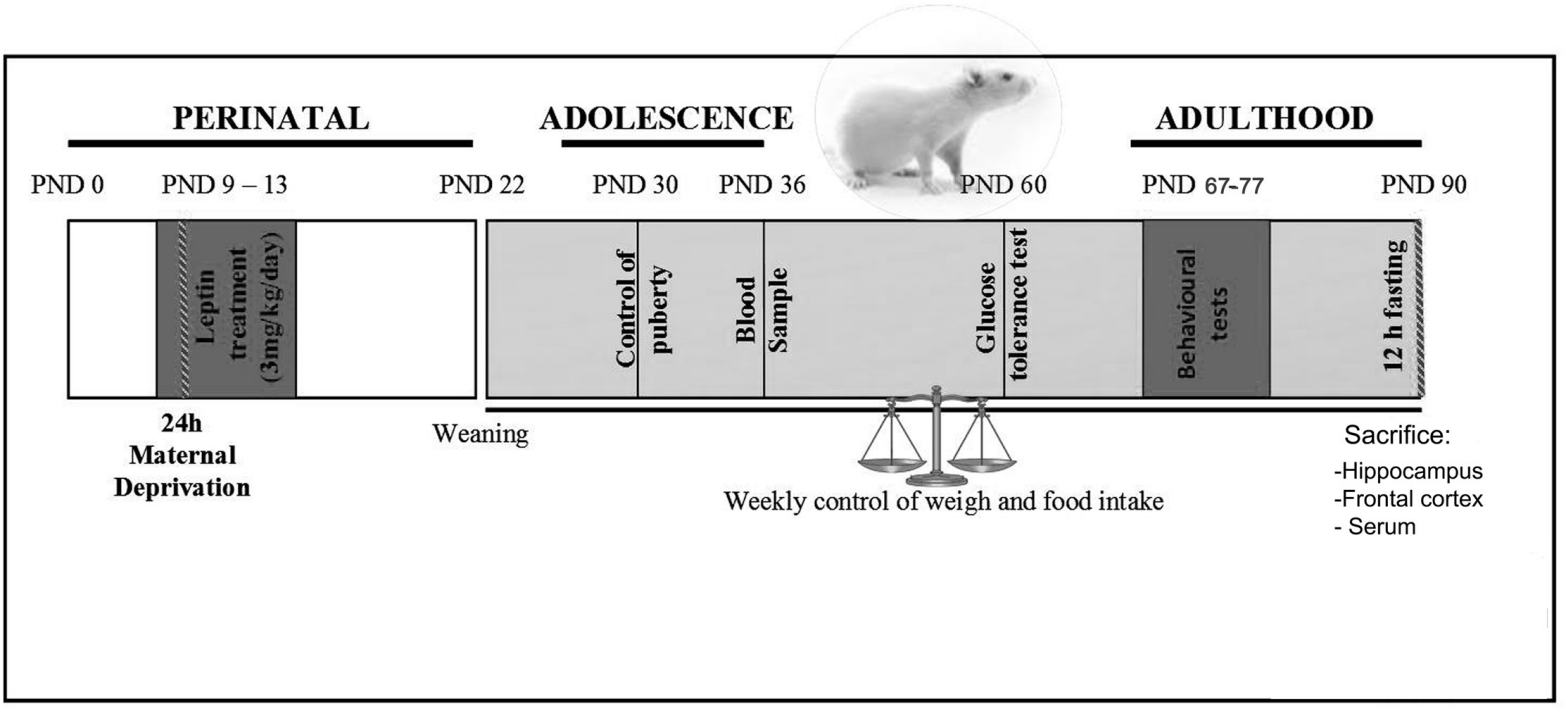
Experimental design diagram.

A summary of the experimental design can be seen in Fig 1. Maternal deprivation was performed as previously described [13]. Briefly, beginning at 09:00 on PND9, mothers from the deprived group were removed and placed in a cage beside the home cage in the same room. On PND10, mothers were returned to the cage of their respective litters. Mothers of the control litters were left undisturbed.

### Leptin treatment

From PND9 until PND13 rats were injected *sc* with 3 mg/kg bodyweight (bw) of rat leptin (National Hormone& Pituitary Program, Torrance, CA). This dose was chosen on the basis of a previous study by our own group [41]. The animals received one injection per day at 9:00. Control rats were injected with the same volume (2.5 ml/kg) of vehicle (saline + 0.1% BSA). After all the pups of the litter were injected they were returned to their mother. All rats were left undisturbed until they were weaned at PND22, and at this time they were separated and placed 2 rats of the same sex and experimental group/cage. Each experimental group consisted of 12 animals from three different litters (4 animals/litter).

### Behavioral testing

All animals were submitted to the behavioral tests, and only the animals that did not fulfill the test requirements were excluded from the statistical analyses.

#### Open field test (OF)

The open field test (OF) was performed in a square arena (60 cm x 60 cm x 45 cm) with matte-painted metallic walls and a plastic-covered wooden floor divided into 36 squares (10 cm x 10 cm) by white painted lines. On PND67 the animal’s general activity was evaluated by video-recording (Sony DCR-DVD310E) observation as open field (OF) test. Total ambulation (horizontal motor activity) was calculated as peripheral (by the walls) and internal (by the squares not adjacent to the walls) line crossings. Rearing frequency (vertical motor activity) and percentage of internal ambulation, considered as an index of emotionality [42] and calculated as the percentage of internal ambulation refereed to total ambulation [(internal line crossings/total line crossings) x 100], were also analyzed.

For the statistical analyses we used between 10–12 males and females per experimental group from 3 different litters (3–4 animals/litter/experimental group). Two MDLept males and 2 CoLept females were excluded from the statistical analyses because there were some troubles with videotape recorder.

#### Novel object test (NOT)

The novel object test (NOT) was performed in the same apparatus used for OF. The test was performed as previously described [43] with some minor modifications [44]. Animals were allowed to freely explore the arena, under dim light conditions, for 5 min during 3 consecutive days (habituation period). On the test day (PND70), rats were first exposed to two identical objects (two plastic boxes) during a 3 min training session. Rats were then exposed, following a 4 h inter-trial interval, to one of the previously encountered objects (familiar object, F1 or F2) and to a novel, non-familiar object (metallic colored box, N) in the test session. Objects were not bigger than twice the size of a rat, and were located in contiguous corners, at a distance of 10cm from the walls. For each animal, the positions of the objects were not changed between the training and the test session. However, the objects’ positions were changed between animals in order to avoid spatial preference.

Both training and test sessions were video recorded (Sony DCR-DVD310E), and animal’s behavior was later evaluated by an experienced observer by means of an event-recorder software (The Observer XT 11, Noldus, Wageningen, Netherlands). Time spent exploring the objects during the two sessions was registered. Exploration of an object was considered whenever animals pointed their nose toward an object at a maximum distance of 1 cm, whereas turning around, climbing and/or biting the objects were not considered as exploration [45]. In the test session, the discrimination index (DI) was calculated as the difference between the time spent exploring the novel object (N) and the familiar one (F1 or F2) in relation to the total time spent exploring the objects [(N - F)/(N + F)]. Animals that explored the two objects less than 10 s during the training and/or the test session were excluded from the statistical analysis, as were animals that explored one of the objects less than 1 s during the test session. Since an animal’s capacity to discriminate between the novel and the familiar objects diminishes with time [45, 46], and in the present conditions animals explored the objects during the first minute more than half the time spent in exploration during the whole testing session, data from the first minute of the test session were employed for the statistical analyses.

For the statistical analyses we used between 8–12 males and females per experimental group from 3 different litters (3–4 animals/litter/experimental group). Two CoLept males, 1 MDVh male, 4 MDLept males, and 2 CoLept females were excluded from the statistical analyses since they did not fulfill the test requirements.

#### Elevated plus-maze (EPM)

The elevated plus-maze (EPM) was formed by two open arms (50 cm x 10 cm) and two equally sized enclosed arms with 40 cm high walls, arranged so that the arms of the same type are opposite to each other. The junction of the four arms formed a central square area (10 cm x 10 cm). The apparatus was made of black hard plastic material and elevated to a height of 62 cm. On PND77, animals were allowed to freely explore the maze for 5 min under dim red light conditions. Frequency and duration of open and closed arm visits were separately recorded, with an arm visit being considered whenever an animal entered it with their four limbs. Percentages of open arm entries and time spent in the open arms were calculated as referred to total arm entries and total time in arms, respectively, and were considered as the most relevant parameters related to anxiety. In turn, closed arm entries were considered as an index of general motor activity [47, 48]. Animals that fall off the EPM were not included in the statistical analysis. The animals that fall off the maze were distributed as follows: males: CoVh (3), CoLept (6), MDVh (3), MDLept (4); females: CoVh (1), CoLept (2), MDVh (7) and MDLept (7). Although we did not record the risk taking behaviors, we did observe that the animals that fall of the maze showed a high frequency of this type of behaviors such as risk assessment with more than a half of the body out of the open arms (especially MD females). Given the high frequency of these patterns, particularly in MD females, we analyzed the frequency of falls from the EPM by a chi-squared test (see results). As after excluding the animals fallen from the maze, in MD female groups there were no enough animals from different litters to perform an ANCOVA, we decided to carry out this analyses only in males.

### Tissue collection

On PND90 all rats were sacrificed after a 12h fast by rapid decapitation and trunk blood was collected in tubes and rapidly placed on ice and kept overnight at 4°. The blood was centrifuged (3000 rpm for 15 min) and the serum collected and stored at −20° until processed and the brain was rapidly removed. The hippocampus and frontal cortex were dissected. They were then frozen in liquid nitrogen and stored at −80° until analyses. To avoid regional variability, the left and right sides were alternated in both brain areas and for both protein and mRNA determinations.

### Western Blotting

Tissue from six males and females per experimental group (2 animals/ litter/group), randomly selected, was homogenized on ice in 300μl of radioimmunoprecipitation assay lysis buffer (RIPA) with an EDTA-free protease inhibitor cocktail (Roche Diagnostics). After homogenization, samples were left overnight at -80°C. The following day they were centrifuged at 14,000 rpm for 20 min at 4°C. Supernatants were transferred to a new tube and the protein concentration was estimated by Bradford protein assay.

In each assay the same amount of protein was loaded in all wells (10, 20 or 40 μg) depending on the protein to be detected and resolved by using 8–12% SDS-acrylamide gels. After electrophoresis proteins were transferred to polyvinylidine difluoride (PVDF) membranes (Bio-Rad, Hercules, CA, USA) and transfer efficiency was determined by Ponceau red dyeing. Membranes were then blocked with Tris-buffered saline (TBS) containing 5% (w/v) bovine serum albumin (BSA; Sigma-Aldrich, Schnelldorf, Germany) and incubated with the appropriate primary antibody. The antibodies employed included anti-NG2 (Sigma-Aldrich, St Louis, MO, USA), anti-glial fibrillary acidic protein (GFAP; Sigma-Aldrich, St Louis, MO, USA), anti-PSD95 (Thermo Scientific, Rockford, IL, USA), anti-neural cell adhesion molecule (NCAM; Millipore, Temecula, CA, USA), anti-vimentin (Sigma-Aldrich, St. Louis, MO, USA) and anti-β actin (Thermo Scientific, Cheshire, UK) were used at a concentration of 1:1000; anti-neuronal nuclei (NeuN; Millipore, Temecula, CA, USA), anti-cannabinoid receptor type 1 (CB1; Sigma-Aldrich, St Louis, MO, USA), anti-brain-derived neurotrophic factor (BDNF; Sta. Cruz biotechnology, Dallas, TX, USA) and anti-glutamate aspartate transporter (GLAST; Alpha Diagnostic International, S. Antonio, TX, USA) were used at a concentration of 1:500; and anti-synaptophysin (Sigma-Aldrich, St Louis, MO, USA) was used at a concentration of 1:2000.

Membranes were subsequently washed and incubated with the corresponding secondary antibody conjugated with peroxidase (1:2000; Pierce, Rockford, IL, USA). Bound peroxidase activity was visualized by chemiluminescence and quantified by densitometry using an ImageQuant LAS4000 mini TL Software (GE Healthcare Europe GmbH, Spain). All blots were rehybridized with actin to normalize each sample for gel loading variability. All data are normalized to control values on each gel.

For the statistical analyses we used between 5–6 males and females per experimental group from 3 different litters (1–2 animals/litter/experimental group) and, in some cases, this number of animals was adjusted to the amount of available sample.

### Quantitative real-time PCR

Total RNA was extracted from the hippocampus and frontal cortex (alternating the left and right side in both brain areas to avoid variability) of 6 males and females per experimental group (2 animals/ litter/group), randomly selected, by using TRIzol® Reagent (Invitrogen). High Capacity cDNA Reverse Transcription kits (Applied Biosystems, Foster City, CA) were used according to the manufacturer's protocol on a Peltier thermal Cycler Tetrad2 (BioRad) to transcribe 2 μg total RNA isolated from each tissue.

Amplification of the cDNA template was performed with an ABI PRISM 7900HT sequence Detection System (Applied Biosystems) using TaqMan Universal PCR Master Mix (Applied Biosystems) and TaqMan Gene Expression Assay kits for each detected gene (Applied Biosystems). The commercial reference for each predesigned expression assay is as follows for each gene measured in the hippocampus and frontal cortex: corticotropin-releasing hormone (CRH, Rn01462137_m1), leptin receptor (LepR; Rn01433250_m1), insulin-like growth factor 1 (IGF1, Rn99999087_m1) and insulin-like growth factor 1 receptor (IGF1R, Rn01477918_m1). Results were normalized to actin (Rn00667869_m1) mRNA levels for both tissues.

According to manufacturer's guidelines, the ΔΔCT method was used for relative quantification. Statistics were performed using ΔCT values.

For the statistical analyses we used between 5–6 males and females per experimental group from three different litters (1–2 animals/litter/experimental group). Samples that did not meet the standards of purity and /or integrity were excluded

### Leptin assay

Leptin was measured in duplicate by a multiplexed magnetic bead immunoassay kit (Millipore Corporation), as previously described [49]. Briefly, beads conjugated to the appropriate antibody and serum samples (25 μl each) were incubated over night at 4°C with shaking. Wells were washed three times using a wash buffer and antibody conjugated to biotin (50 μl) was added. After incubation for 30 min at room temperature with shaking, beads were incubated during 30 min with 50 μl streptavidin conjugated to phycoerythrin. Beads were analyzed in the Bio-Plex suspension array system 200. Raw data (mean fluorescence intensity) were analyzed using the Bio-Plex Manager Software 4.1 (Bio-Rad Laboratories). For the statistical analyses we used 6 males and 6 females per experimental group from three different litters (2 animals/litter/experimental group), randomly selected.

### Statistical analysis

In the case of the parameters measured by Western blotting or RT-qPCR, the usual criterion to reduce litter effect in forming experimental groups, i.e., a maximum of 2 male and 2 female offspring from any given litter was fulfilled. Thus, for the analyses of the molecular parameters, 3-way ANOVA was used (with the 3 factors being: sex, MD and Leptin treatment), followed by 2-way ANOVA where appropriate i.e. in the presence of significant interaction between main factors in the 3-way ANOVA. As in the behavioral tests there were more than 2 males/females from the same litter in each experimental group, in this case we have controlled for litter effects by performing ANCOVA analyses (including “litter” as covariate). Normality was checked by Shapiro-Wilks’s test (p>0.05). The criterion of normally distributed data were not always met in some populations under study and, so, when necessary, data were transformed by the Neperian logarithm function, aiming to satisfy the assumption of normality for ANOVA. The Tukey test was performed for post-hoc comparisons except when the criterion of homoscedasticity (Levene test, p>0.05) was not met, in this case another more restrictive post-hoc comparison test (Bonferroni test), was used to avoid a type I error. In the EPM, the frequency of events (falls from the EPM) was analyzed with a chi-squared test. In all cases, P < 0.05 was considered statistically significant. All statistical analyses were carried out with SPSS, version 19.0 (SPSS Inc., Chicago, IL, USA).

## Results

### Behavioral testing

**Fig 2:**
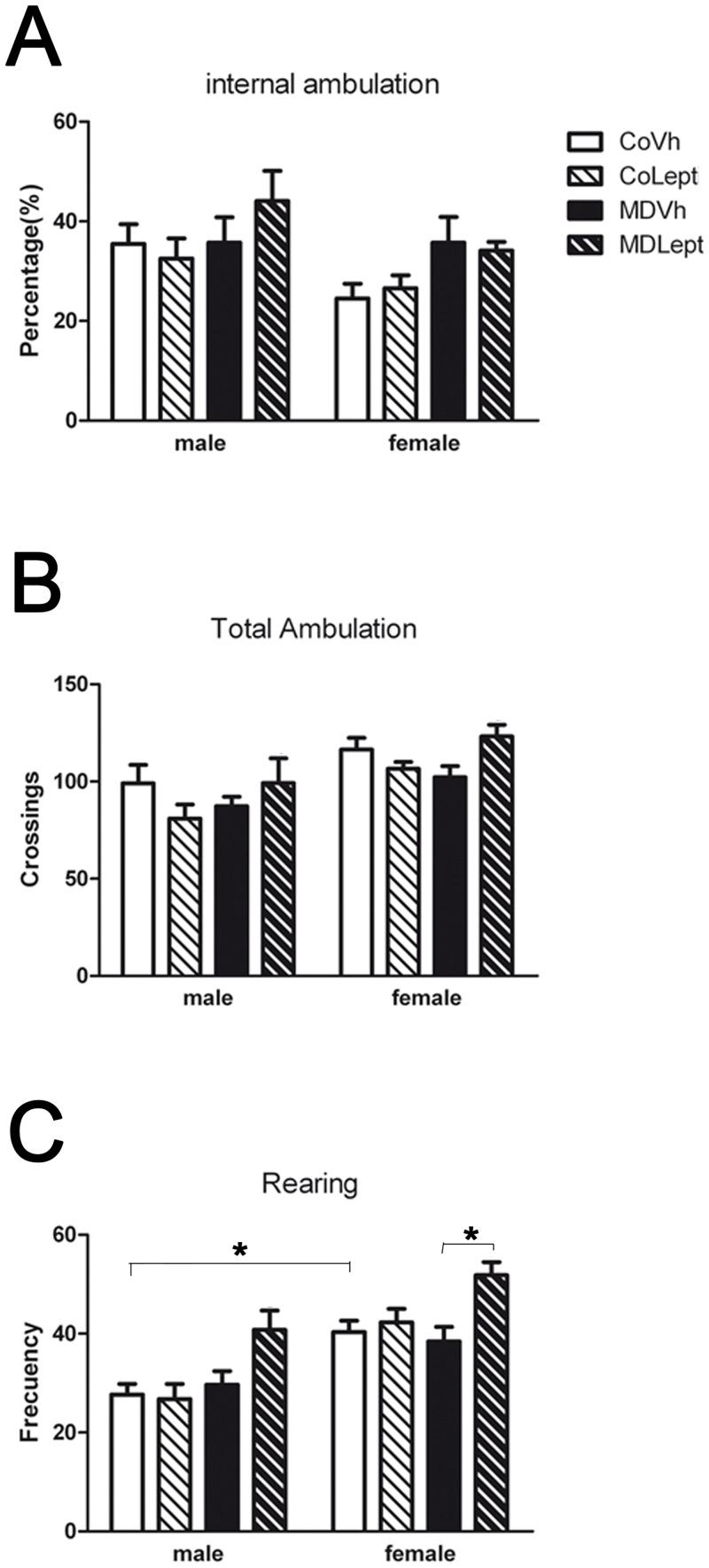
Open field test. (A) Emotionality index, calculated as the percentage of internal ambulation referred to total ambulation, (B) total ambulation and (C) rearing frequency were registered in maternally deprived (MD) or control (Co) male and female rats treated with leptin (Lept) or vehicle (Vh) from postnatal day (PND) 9 until PND13 (n = 10–12). Post hoc test: *<0.05.

**Fig 3:**
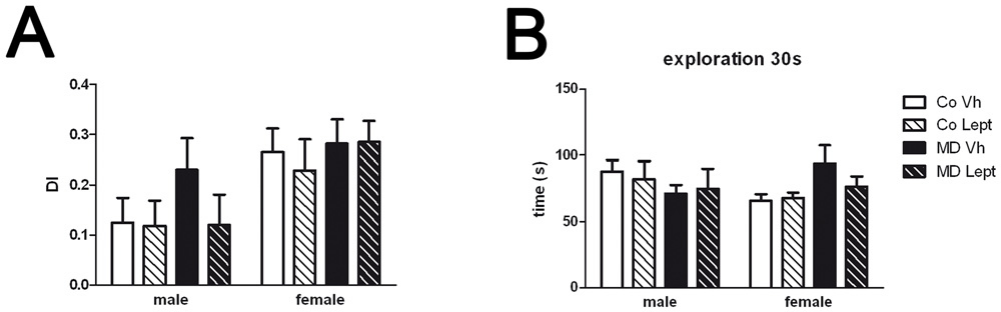
Novel object test (NOT). (A)Discrimination index (DI) and (B) time spent in exploration of the object during the 30 second training session of maternally deprived (MD) or control (Co) male and female rats treated with leptin (Lept) or vehicle (Vh) from postnatal day (PND) 9 until PND13 (n = 8–12).

Main statistical results are shown in Table 1 and means ± SEMs are represented as histograms in Figs 2 and 3.

**Table 1:** Main statistical results corresponding to behavioral tests.

	%internal ambulation (OF)		Total ambulation (OF)		Rearing frequency (OF)		DI (NOT)		Exploration 30 s (NOT)		%open arms entries (EPM)		Closed arms entries (EPM)	
	F_1,83_	p-value	F_1,83_	p-value	F_1,83_	p-value	F_1,78_	p-value	F_1,78_	p-value	F_1,25_	p-value	F_1,25_	p-value
**Litter**	**9.29**	**<0.005**	**0.22**	**ns**	**4.09**	**<0.05**	**4.62**	**<0.05**	**4.88**	**<0.05**	**0.20**	**ns**	**8.03**	**<0.01**
**Sex**	**6.01**	**<0.05**	**15.21**	**<0.005**	**37.95**	**<0.005**	**8.78**	**<0.01**	**0.09**	**ns**	**-**	**-**	**-**	**-**
**MD**	**12.70**	**<0.005**	**0.31**	**ns**	**4.80**	**<0.05**	**3.48**	**ns**	**0.73**	**ns**	**0.19**	**ns**	**0.50**	**ns**
**Leptin**	**5.67**	**<0.05**	**0.23**	**ns**	**1.33**	**ns**	**0.40**	**ns**	**0.59**	**ns**	**2.61**	**ns**	**1.89**	**ns**
**Sex x MD**	**0.31**	**<0.05**	**0.05**	**ns**	**1.06**	**ns**	**0.04**	**ns**	**4.41**	**<0.05**	**-**	**-**	**-**	**-**
**Sex x Leptin**	**0.27**	**ns**	**0.67**	**ns**	**0.50**	**ns**	**0.27**	**ns**	**0.03**	**ns**	**-**	**-**	**-**	**-**
**MD x Leptin**	**0.31**	**ns**	**8.34**	**<0.01**	**9.47**	**<0.005**	**0.53**	**ns**	**0.32**	**ns**	**0.94**	**ns**	**0.65**	**ns**
**Sex x MD x Leptin**	**1.97**	**ns**	**0.01**	**ns**	**0.01**	**ns**	**1.35**	**ns**	**0.71**	**ns**	**-**	**-**	**-**	**-**

Main significant results extracted from the three-way ANCOVA (litter was considered as a covariate factor). See material and methods section for details.

Despite the litter significantly affected some of the behavioral parameters studied (Table 1), the effects of our experimental factors (sex, MD, and leptin administration) remained significant in most cases. Taken together, the significant effects here reported are confirmed since they remain significant when correcting by the litter. The ANCOVA performed on the parameters measured in the open field test revealed a significant effect of sex, with females showing the lowest value for the percentage of internal ambulation; and the highest value for total ambulation and rearing frequency. A main effect of MD on the percentage of internal ambulation was found, with MD rats showing an increase in this value. Leptin treatment significantly increased total ambulation and rearing frequency in MD rats (Fig 2).

In the elevated plus maze, where only males were analyzed by the ANCOVA (as indicated in Material and Methods), the analysis did not reveal any significant difference among male groups for % of open arm entries (CoVh: 52.00±6.14; CoLept: 33.38±8.39; MDVh: 45.04±3.56; MDLept: 53.37±5.95; n = 6–9) or closed arm entries (CoVh: 9.44±0.84; CoLept: 8.17±0.75; MDVh: 8.56±1.00; MDLept: 8.83±0.60; n = 6–9). It is worth mentioning that in this test, MD females fall more often from the apparatus than MD males (χ^2^_(1)_ = 6.75, p<0.01), with 14 out of 24 MD females falling from the EPM as opposed to 7 out of 22 MD males.

In the novel object test, the analyses of the discrimination index, which indicates the working memory of the animals in the novel object test did not reveal any significant effect. A main effect of sex on the discriminatory index (DI) was found, with females showing higher values than males (Fig 3).

### Synaptic plasticity markers. Protein levels

**Fig 4:**
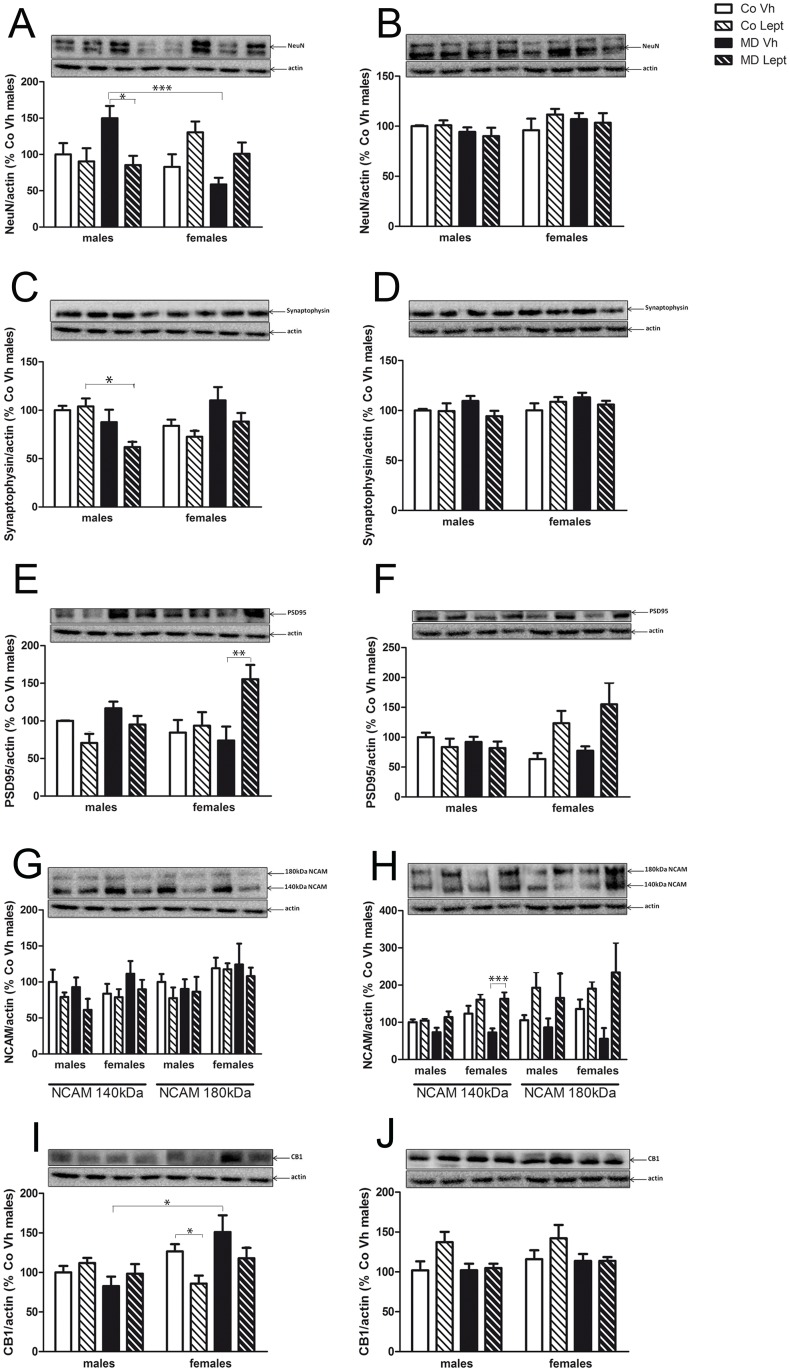
Protein levels in the hippocampus (left column) and frontal cortex (right column) of NeuN (A & B), synaptophysin (C & D), PSD95 (E & F), NCAM (G & H) and CB1 receptor (I & J) in maternally deprived (MD) or control (Co) male and female rats treated with leptin (Lept) or vehicle (Vh) from postnatal day (PND) 9 until PND13 (n = 5–6). Representative western blotting immunoassays of each protein from tissue homogenates of each brain area are presented above each histogram. Post hoc test: *<0.05, **<0.01, ***<0.005.

Main results obtained in the 3-Way ANOVA are shown in Table 2, and Fig 4 represent mean ± SEM for the different experimental groups as well as significant differences derived from post hoc comparisons.

**Table 2:** Main statistical results corresponding to synaptic plasticity markers (protein levels).

HIPPOCAMPUS	NeuN	Synaptophysin	PSD95	NCAM(140kDa)	NCAM(180kDa)	BDNF	CB1
	**F**_**1,35**_ **(p-value)**	**F**_**1,33**_ **(p-value)**	**F**_**1,35**_ **(p-value)**	**F**_**1,39**_ **(p-value)**	**F**_**1,36**_ **(p-value)**	**F**_**1,40**_ **(p-value)**	**F**_**1,37**_ **(p-value)**
**Sex**	**1.54 (ns)**	**0.01 (ns)**	**0.34 (ns)**	**0.61 (ns)**	**5.84 (<0.05)**	**0.06 (ns)**	**6.36 (<0.05)**
**MD**	**0.04 (ns)**	**0.25 (ns)**	**3.47 (ns)**	**0.12 (ns)**	**0.04 (ns)**	**0.11 (ns)**	**0.09 (ns)**
**Leptin**	**0.14 (ns)**	**5.02 (<0.05)**	**0.31 (ns)**	**4.12 (<0.05)**	**1.07 (ns)**	**0.05 (ns)**	**1.24 (ns)**
**Sex x MD**	**5.36 (<0.05)**	**15.38 (<0.005)**	**0.12 (ns)**	**2.67 (ns)**	**0.01 (ns)**	**0.18 (ns)**	**7.87 (<0.01)**
**Sex x Leptin**	**14.84 (<0.005)**	**0.22 (ns)**	**11.61 (<0.005)**	**0.45 (ns)**	**0.07 (ns)**	**0.30 (ns)**	**9.81 (<0.005)**
**MD x Leptin**	**2.01 (ns)**	**2.68 (ns)**	**2.17 (ns)**	**0.51 (ns)**	**0.03 (ns)**	**0.01 (ns)**	**0.53 (ns)**
**Sex x MD x Leptin**	**1.37 (ns)**	**0.61 (ns)**	**2.55 (ns)**	**0.03 (ns)**	**0.63 (ns)**	**1.01 (ns)**	**0.10 (ns)**
**FRONTAL CORTEX**	**F**_**1,40**_ **(p-value)**	**F**_**1,40**_ **(p-value)**	**F**_**1,34**_ **(p-value)**	**F**_**1,37**_ **(p-value)**	**F**_**1,35**_ **(p-value)**	**F**_**1,35**_ **(p-value)**	**F**_**1,40**_ **(p-value)**
**Sex**	**2.63 (ns)**	**2.76 (ns)**	**0.31 (ns)**	**9.55 (<0.005)**	**1.10 (ns)**	**3.89 (ns)**	**2.03 (ns)**
**MD**	**0.46 (ns)**	**0.97 (ns)**	**0.41 (ns)**	**2.50 (ns)**	**3.48 (ns)**	**0.01 (ns)**	**3.25 (ns)**
**Leptin**	**0.20 (ns)**	**0.98 (ns)**	**3.31 (ns)**	**17.58 (<0.005)**	**17.71 (<0.005)**	**0.52 (ns)**	**4.96 (<0.05)**
**Sex x MD**	**0.95 (ns)**	**0.15 (ns)**	**0.94 (ns)**	**0.55 (ns)**	**0.18 (ns)**	**0.06 (ns)**	**0.03 (ns)**
**Sex x Leptin**	**0.59 (ns)**	**1.32 (ns)**	**11.76 (<0.005)**	**4.03 (ns)**	**0.89 (ns)**	**0.08 (ns)**	**0.35 (ns)**
**MD x Leptin**	**1.48 (ns)**	**4.20 (<0.05)**	**0.01 (ns)**	**4.64 (<0.05)**	**0.32 (ns)**	**0.07 (ns)**	**3.40 (ns)**
**Sex x MD x Leptin**	**0.48 (ns)**	**0.01 (ns)**	**0.14 (ns)**	**0.17 (ns)**	**0.85 (ns)**	**0.14 (ns)**	**0.13 (ns)**

Main significant results extracted from the three-way ANOVA. See material and methods section for details.

#### NeuN

As Table 2 shows, the 3-Way ANOVA for hippocampal NeuN revealed two significant double interactions sex x MD and MD x leptin. Two-way ANOVA split by MD revealed a main effect of sex [F(1,18) = 7.87; p<0.05] and a double interaction between sex and leptin treatment [F(1,18) = 15.71; p<0.005] in MD rats, without effects in Co groups. As Fig 4A show, MDVh males tended to increase NeuN levels when compared to CoVh group (Tukey’s post hoc, p = 0.1), and the leptin treatment reversed this trend, i.e., significantly decreased NeuN levels of MD males to control values (Tukey post hoc, p<0.05). Also, the figure shows a sexual dimorphism between MDVh with females showing the lowest values (Tukey post hoc, p<0.005).

No significant differences were found in the frontal cortex (Fig 4B)

#### Synaptophysin

The 3-Way revealed a significant effect of the leptin treatment as well as significant sex MD interaction on the expression of hippocampal synaptophysin. Two-way ANOVA split by sex revealed a main effect of MD in males [F(1,17) = 11.14; p<0.005] and in females [F(1,16) = 5.16; p<0.05] with MD males showing lower levels and MD females higher levels than their respective control groups. Post hoc comparisons (Fig 4C) only revealed a significant decrease in MDLept males comparing with CoLept males (Tukey post hoc, p<0.05).

No main effects were found in the frontal cortex (Fig 4D). However, there was a significant interaction between MD and leptin treatment. Two-way ANOVA split by MD revealed an effect of leptin treatment in MD (but not in Co) animals [F(1,20) = 5.72; p<0.05] As Fig 4D shows, MDLept animals had lower synaptophysin levels than MDVh animals.

#### PSD95

The 3-way ANOVA for hippocampal PSD95 levels showed a significant interaction between sex and leptin. Two-way ANOVA split by sex revealed an effect of leptin treatment in both sexes (males: [F(1,19) = 6.65; p<0.05]; females: [F(1,16) = 5.22; p<0.05]). As Fig 4E shows, the leptin effect had an opposite direction in males and females, i.e., in males, leptin modestly decreased PSD95 expression whereas in females leptin increased this parameter. Post hoc comparisons showed a significant difference between MDVh and MD Lept females (Tukey’s post hoc, p<0.01).

The results obtained for frontal cortex were similar to those of hippocampus. The 3-Way ANOVA revealed a significant sex x leptin interaction and the 2-Way ANOVA split by sex revealed a significant effect of leptin [F(1,17) = 9.92; p<0.05] only in females. As Fig 4F shows, as in hippocampus, MD females treated with leptin showed the highest PSD95 levels.

#### NCAM

The 3-Way ANOVA for the two forms of NCAM in the hippocampus (Fig 4G) revealed a significant effect of leptin for 140 kDa NCAM expression, with leptin treated animals showing a decreased expression of this protein. In the case of 180 kDa NACM, a main effect of sex was observed with females showing higher values than males (Fig 4G).

In the frontal cortex (Fig 4H) a significant effect of leptin was found for both isoforms, so that animals exposed to leptin during the neonatal period showed significantly increased expression of the two proteins. A general effect of sex was found for NCAM 140 kDa, with females showing significantly higher levels than males. Also, in this isoform (NCAM 140 kDa) there was a main effect of MD and a significant MD x leptin interaction. The Two-way ANOVA split by MD revealed an effect of leptin treatment (an increase in protein expression) only in MD but not in control animals [F(1,18) = 19.71; p<0.005]. Post hoc comparisons showed a significant difference between MDLept and MDVh females (Tukey’s post hoc, p<0.005).

#### BDNF

The analysis of BDNF expression in the hippocampus [Males (CoVh: 100.0±9.2; CoLept: 92.7±12.1; MDVh: 87.9±5.3; MDLept: 94.6±10.8) and Females (CoVh: 93.1±10.5; CoLept: 97.1±4.6; MDVh: 100.3±9.9; MDLept: 91.1±10.9)] and in the frontal cortex [Males (CoVh: 100.0±15.1; CoLept: 105.3±14.8; MDVh: 102.3±14.1; MDLept: 105.6±4.2) and Females (CoVh: 122.6±17.7; CoLept: 126.1±6.0; MDVh: 112.8±16.3; MDLept: 128.8±15.6)] did not reveal any significant difference.

#### CB1 cannabinoid receptor

The 3-Way ANOVA for hippocampal CB1 receptor expression revealed a main effect of sex and, as Fig 4I shows, there was a sex difference between MDVh, males and females, with females showing the higher values (Tukey’s post hoc, p<0.05). The 3-Way ANOVA also revealed significant interactions between sex and MD and sex and leptin. Two-way ANOVA split by sex revealed main effects of MD [F(1,17) = 4.67; p<0.05] and leptin treatment [F(1,17) = 8.69; p<0.01] only in females, and post hoc comparisons showed a significant difference between CoVh and Co Lept (Tukey´s post hoc test, p<0.05), with these latter females showing the lowest values (Fig 4I).

In the frontal cortex, the 3-Way ANOVA only revealed a main effect of leptin, with this effect being observable only in control non deprived animals (Fig 4J).

In summary, in the parameters described in this section, the hippocampus appeared to be more susceptible to the effects of the treatments than the frontal cortex and the vast majority of the effects were sex-dependent. Hippocampal NeuN, synaptophysin and CB1 were affected by both MD and leptin, whereas PDD95 and NCAM were mainly affected by the neonatal leptin treatment. In the frontal cortex the parameter most affected by the treatment was NCAM.

### mRNA levels

**Fig 5:**
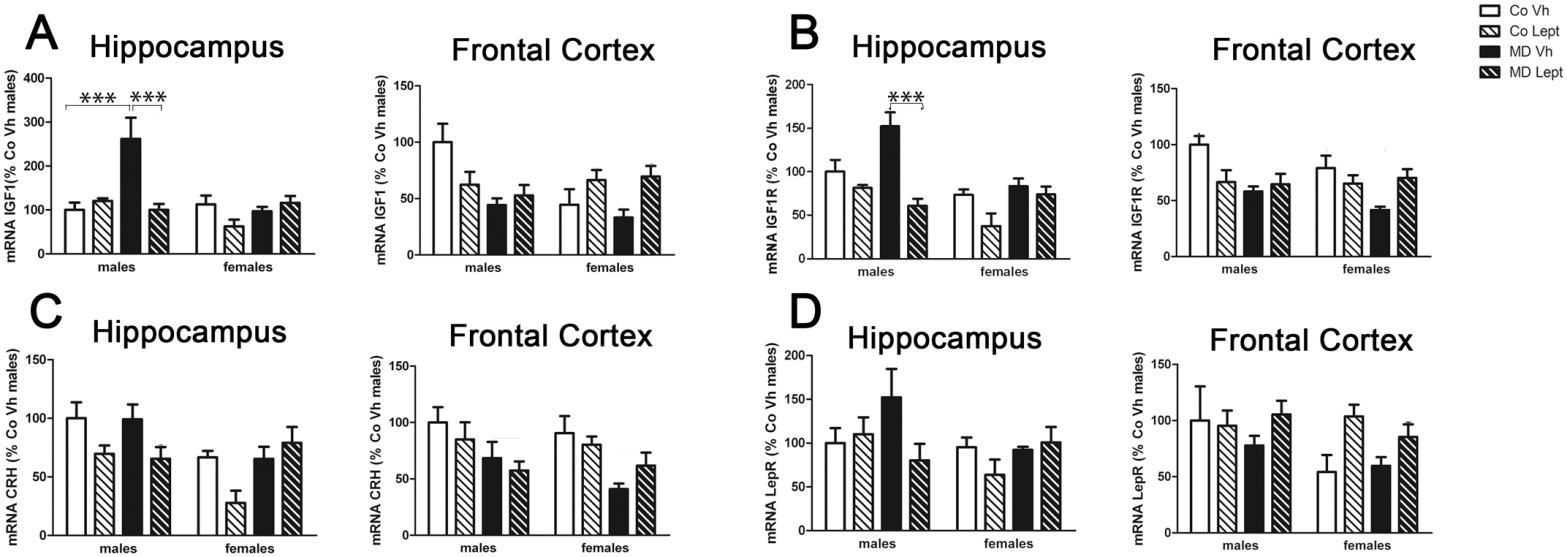
Expression levels in the hippocampus and frontal cortex of the mRNAs encoding insulin-like growth factor 1 (IGF1) (A), IGF1 receptor (IGF1R) (B), corticotropin-releasing hormone (CRH) (C) and leptin receptor (LepR) (D) in maternally deprived (MD) or control (Co) male and female rats treated with leptin (Lept) or vehicle (Vh) from postnatal day (PND) 9 until PND13 (n = 5–6). Post hoc test: ***p<0.005.

Main results obtained in the 3-Way ANOVA are shown in Table 3 and Fig 5 represent mean ± SEM for the different experimental groups as well as significant differences derived from post hoc comparisons.

**Table 3:** Main statistical results corresponding to mRNA levels.

HIPPOCAMPUS	IGF1	IGF1R	CRH	LepR
	**F**_**1,37**_ **(p-value)**	**F**_**1,34**_ **(p-value)**	**F**_**1,37**_ **(p-value)**	**F**_**1,39**_ **(p-value)**
**Sex**	**8.66 (<0.01)**	**8.24 (<0.01)**	**9.63 (<0.005)**	**3.14 (ns)**
**MD**	**4.91 (<0.05)**	**1.97 (ns)**	**2.14 (ns)**	**1.23 (ns)**
**Leptin**	**1.53 (ns)**	**15.17 (<0.005)**	**8.45 (<0.01)**	**2.74 (ns)**
**Sex x MD**	**0.01 (ns)**	**1.97 (ns)**	**3.23 (ns)**	**0.05 (ns)**
**Sex x Leptin**	**0.01 (ns)**	**2.40 (ns)**	**1.60 (ns)**	**0.58 (ns)**
**MD x Leptin**	**1.28 (ns)**	**2.51 (ns)**	**2.60 (ns)**	**0.68 (ns)**
**Sex x MD x Leptin**	**14.59 (<0.005)**	**5.40 (<0.05)**	**3.33 (ns)**	**5.69 (<0.05)**
**FRONTAL CORTEX**	**F**_**1,35**_ **(p-value)**	**F**_**1,36**_ **(p-value)**	**F**_**1,38**_ **(p-value)**	**F**_**1,34**_ **(p-value)**
**Sex**	**2.30 (ns)**	**2.18 (ns)**	**1.04 (ns)**	**3.21 (ns)**
**MD**	**5.95 (<0.05)**	**11.20 (<0.005)**	**14.16 (<0.005)**	**0.35 (ns)**
**Leptin**	**0.93 (ns)**	**0.29 (ns)**	**0.01 (ns)**	**5.41 (<0.05)**
**Sex x MD**	**3.59 (ns)**	**0.26 (ns)**	**0.36 (ns)**	**0.01 (ns)**
**Sex x Leptin**	**8.45 (<0.01)**	**3.42 (ns)**	**1.74 (ns)**	**1.52 (ns)**
**MD x Leptin**	**4.10 (<0.05)**	**13.10 (<0.005)**	**1.21 (ns)**	**0.04 (ns)**
**Sex x MD x Leptin**	**1.10 (ns)**	**0.02 (ns)**	**0.44 (ns)**	**1.77 (ns)**

Main significant results extracted from the three-way ANOVA. See material and methods section for details.

#### IGF1 and IGF1R

The 3-Way ANOVA for IGF1 mRNA levels in the hippocampus revealed main effect of sex and MD as well as a significant triple interaction sex x MD x. As Fig 5A MD significantly increased the expression of IGF1 mRNA in males (Bonferroni’s pot hoc, p<0.005) and the neonatal leptin treatment reversed this effect (Bonferroni’s pot hoc, p<0.005). The analyses of IGF1R mRNA levels in the hippocampus revealed main effects of sex and leptin treatment, as well as a triple interaction sex x MD x leptin. As Fig 5B shows, MD tended to increase IGF1R mRNA levels in males (Tukey’s post hoc, p = 0.057), whereas the leptin treatment counteracted this trend (Tukey’s post hoc, p<0.005).

**Fig 6:**
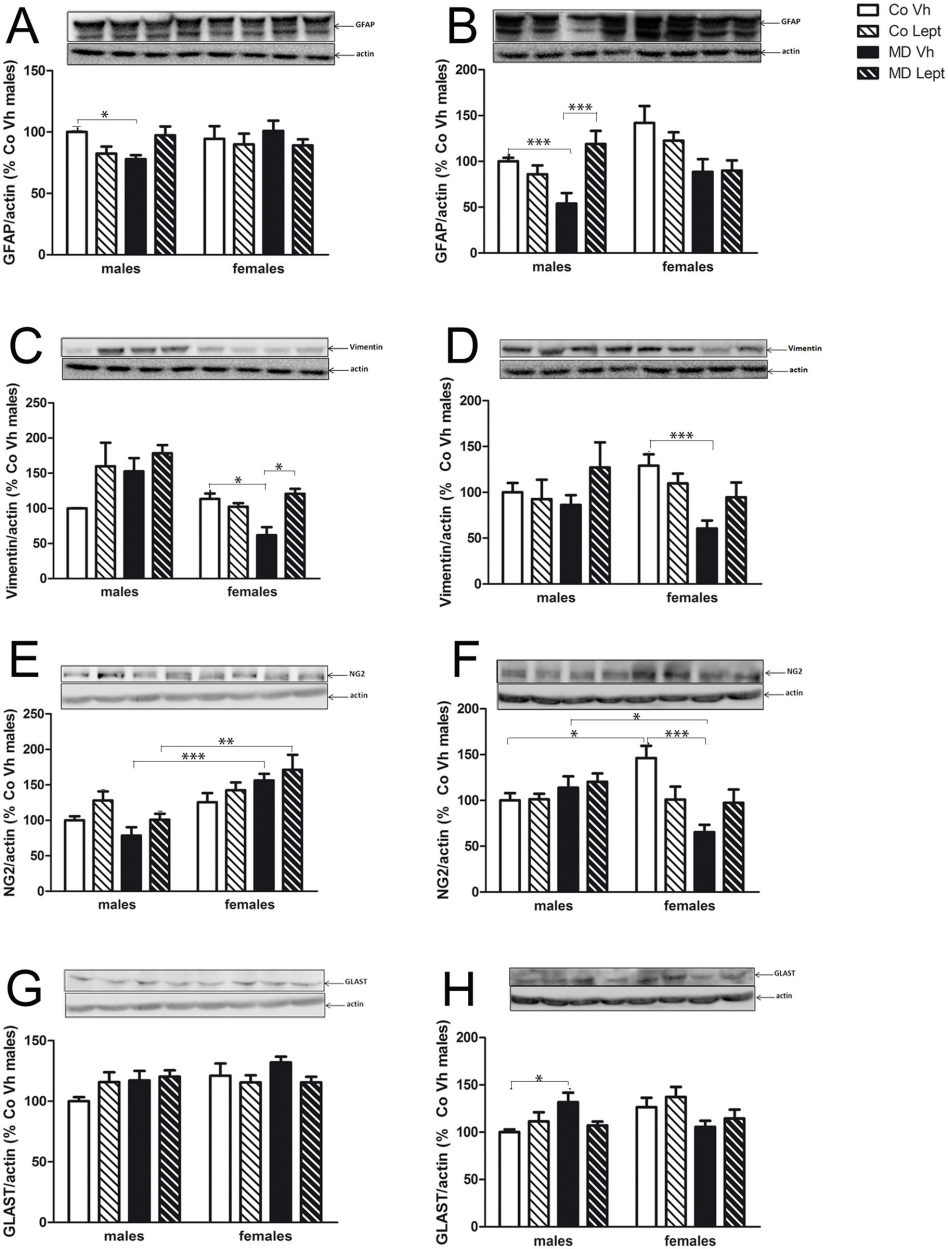
Protein levels in the hippocampus (left column) and frontal cortex (right column) of GFAP (A & B), vimentin (C & D), NG2 (E & F) and GLAST (G & H) in maternally deprived (MD) or control (Co) male and female rats treated with leptin (Lept) or vehicle (Vh) from postnatal day (PND) 9 until PND13 (n = 5–6). Representative western blotting immunoassays of problem protein from tissue homogenates of each brain area are presented above each histogram. Post hoc test: *<0.05, **<0.01, ***<0.005.

In the frontal cortex, the 3-Way ANOVA for IGF1 mRNA levels revealed a main effect of MD and significant interactions between sex x MD and MD x leptin treatment. Two-way ANOVA split by sex showed a significant effect of MD effect in males [F(1,17) = 8.05; p<0.05] and a significant effect of leptin treatment in females [F(1,18) = 8.84; p<0.01]. As Fig 6A shows, MD decreased IGF1 mRNA expression in control non deprived males while females exposed neonatally to leptin showed increased IGF1 mRNA levels. The analyses of IGF1R mRNA levels in the frontal cortex rendered a main effect of MD and a significant interaction between MD and leptin. Two-Way ANOVA split by leptin confirmed that the effect of MD was only significant in the groups treated with vehicle [F(1,20) = 5.06; p<0.05] and not in those receiving the neonatal leptin treatment (Fig 5B)

#### CRH

The 3-Way ANOVA for hippocampal CRH mRNA levels revealed significant main effects of sex and leptin treatment. As a whole, females showed lower CRH mRNA levels than males and the leptin treatment decreased CRH mRNA levels except in MD females (Fig 5C).

As for the frontal cortex, a main effect of MD was found. As Fig 5C shows, MD animals had lower values than control non deprived ones.

#### Leptin receptor

The 3-Way ANOVA for hippocampal leptin receptor (LepR) mRNA showed a triple interaction between sex, MD and leptin treatment but the Bonferroni’ post hoc and Two-way ANOVA split by sex did not reveal any significant effect. In the frontal cortex a main effect of the leptin treatment was found. As Fig 5D shows, leptin treated animals showed increased levels of mRNA LeptR expression, except CoVh animals.

In summary, in the Hippocampus, leptin treatment reversed the effect of MD which increased the levels of IGF1 mRNA in males. In frontal cortex MD decreased IGF1 mRNA and this effect was not reversed by leptin. The pattern of changes of IGF1 was similar to those of IGF1R within each region. In the hippocampus, leptin treatment decreased CRH mRNA expression, whereas in the frontal cortex, MD diminished this parameter. In the frontal cortex, leptin treatment increased leptin receptor mRNA levels

### Protein levels of glial markers

Main results obtained in the 3-Way ANOVA are shown in Table 4 and Fig 6 represent mean ± SEM for the different experimental groups as well as significant differences derived from post hoc comparisons.

**Table 4:** Main statistical results corresponding to glial markers (protein levels).

HIPPOCAMPUS	GFAP	Vimentin	NG2	GLAST
	**F1,36 (p-value)**	**F1,36 (p-value)**	**F1,39 (p-value)**	**F1,40 (p-value)**
**Sex**	**0.69 (ns)**	**0.74 (ns)**	**31.02 (<0.005)**	**2.76 (ns)**
**MD**	**0.01 (ns)**	**0.20 (ns)**	**0.11 (ns)**	**3.12 (ns)**
**Leptin**	**0.53 (ns)**	**0.13 (ns)**	**5.96 (<0.05)**	**0.03 (ns)**
**Sex x MD**	**0.42 (ns)**	**26.86 (<0.005)**	**10.26 (<0.005)**	**0.34 (ns)**
**Sex x Leptin**	**0.81 (ns)**	**0.51 (ns)**	**0.30 (ns)**	**4.83 (<0.05)**
**MD x Leptin**	**2.18 (ns)**	**5.92 (<0.05)**	**0.05 (ns)**	**1.63 (ns)**
**Sex x MD x Leptin**	**4.86 (<0.05)**	**9.79 (<0.005)**	**0.02 (ns)**	**0.01 (ns)**
**FRONTAL CORTEX**	**F1,36 (p-value)**	**F1,37 (p-value)**	**F1,35 (p-value)**	**F1,40 (p-value)**
**Sex**	**6.17 (<0.05)**	**0.08 (ns)**	**0.63 (ns)**	**1.80 (ns)**
**MD**	**8.57 (<0.01)**	**2.23 (ns)**	**2.56 (ns)**	**0.38 (ns)**
**Leptin**	**0.95 (ns)**	**1.29 (ns)**	**0.03 (ns)**	**0.07 (ns)**
**Sex x MD**	**4.64 (<0.05)**	**6.08 (<0.05)**	**13.50 (<0.005)**	**9.26 (<0.005)**
**Sex x Leptin**	**4.16 (<0.05)**	**0.20 (ns)**	**0.42 (ns)**	**1.70 (ns)**
**MD x Leptin**	**8.67 (<0.01)**	**5.77 (<0.05)**	**6.65 (<0.05)**	**2.38 (ns)**
**Sex x MD x Leptin**	**2.98 (ns)**	**0.02 (ns)**	**5.09 (<0.05)**	**2.03 (ns)**

Main significant results extracted from the three-way ANOVA. See material and methods section for details.

#### GFAP

The 3-Way ANOVA showed a significant triple interaction sex x MD x leptin for hippocampal GFAP protein expression. As Fig 6A shows, MD decreased GFAP levels in males (Tukey’s post-hoc, p<0.05) whereas the leptin treatment tended to reverse this effect.

In the frontal cortex, the 3-Way ANOVA showed main effect of sex and MD and the three double interactions were also significant. Two-way ANOVA split by sex revealed an interaction between MD and leptin [F(1,18) = 13.84; p<0.005] in males. As Fig 6B shows, MD significantly decreased GFAP levels in this sex (Tukey’s pot-hoc, p<0.05), whereas the leptin treatment counteracted this effect (Tukey’s pot-hoc, p<0.005). In females, a MD effect was found [F(1,18) = 10.65; p<0.005], with MD females showing decreased GFAP levels.

#### Vimentin

The 3-Way ANOVA for hippocampal vimentin expression revealed the following significant interactions: sex x MD, MD x leptin treatment and sex x MD x leptin. As Fig 6C shows, MD significantly reduced vimentin levels in females (Tukey’s post hoc, p<0.05) whereas the neonatal leptin treatment reversed this effect (Tukey’s post hoc, p<0.05).

The results were similar in the frontal cortex where 3-Way ANOVA showed significant double interactions sex x MD and MD x leptin. Two-way ANOVA split by sex revealed a MD effect [F(1,19) = 12.31; p<0.005] and a double interaction between MD and leptin [F(1,19) = 5.01; p<0.05] in females. As Fig 6D shows, MD significantly reduced vimentin expression in females (Tukey’s post hoc, p<0.005), whereas leptin attenuated this effect.

#### NG2

The 3-Way ANOVA for hippocampal NG2 levels showed main effects of sex (females higher expression than males) and leptin treatment as well as a double interaction between sex and MD. According to this interaction, the 2-Way ANOVA split by sex revealed opposite effects MD in both sexes, a decrease of NG2 levels in males [F(1,20) = 5.86; p<0.05] and an increase in females [F(1,19) = 4.71; p<0.05]. In turn, an effect of the neonatal leptin treatment was found only in males, with treated animals showing an increase on NG2 levels [F(1,20) = 6.31; p<0.05]. As Fig 6E shows, there was a sexual dimorphism in MD animals since MDVh females and MDLept females have higher levels than MDVh (Tukey’s post hoc, p<0.01) and MDLeptin males (Tukey’s post hoc, p<0.005), respectively.

In the frontal cortex, the analysis of NG2 levels revealed a significant triple interaction sex x MD x leptin, as well as two significant double interactions sex x MD and MD x leptin. Fig 6F shows sexual dimorphisms among CoVh and MD Vh animals (Tukey’s post hoc, p<0.05 in both cases). The most interesting result is the effect of MD in females (decreasing NG2 levels) (Tukey’s post hoc, p<0.005) and the fact that leptin treatment attenuated this effect.

#### Glutamatergic receptor

The 3-Way ANOVA performed on the hippocampal levels of GLAST (Fig 6G). only revealed a significant sex x leptin interaction, and the 2-Way ANOVA split by leptin revealed a sex effect [F(1,20) = 6.50; p<0.05] in Vh but not leptin treated groups (females Vh showed higher levels than males Vh). In the frontal cortex, the 3-Way ANOVA revealed a significant interaction between sex and MD. Two-way ANOVA split by sex revealed that, in males, MD increased GLAST expression [F(1,20) = 5.99; p<0.05] whereas neonatal leptin attenuated this effect. On the other hand, MD induced a decrease in GLAST expression in the frontal cortex of females [F(1,20) = 5.29; p<0.05] and this effect was not attenuated by the leptin treatments (see Fig 6H for post hoc significant differences).

In summary, once more the effects observed on glial markers and GLAST were sex dependent. In both regions GFAP expression was decreased by MD in males and this effect was either reversed or attenuated by the neonatal leptin treatment, similar changes were found for vimentin and NG2, but in these cases in females. Cortical GLAST expression was differently affected in males and females.

### Leptin levels

The 3-Way ANOVA for leptin levels showed a significant effect of sex [F(1,40) = 6.27; p<0.05]. Males: CoVh: 4.05±0.87; CoLeptin:3.16±0.76; MDVh:2.99±0.65; MDLeptin:2.88±0.64. Females: CoVh:1.93±0.23; CoLeptin:2.12±0.29; MDVh:2.44±0.56; MDLeptin:1.65±0.31. N = 6 in all groups

## Discussion

The main results of this study can be summarized as follows: 1) MD females appeared to show an increased impulsivity or reduce “fear” 2) As opposed to previous studies, MD did not cause a deficit in working memory, 3) Neonatal leptin treatment reversed or attenuated the effects of MD in some of the parameters analyzed (e.g., mRNA expression of hippocampal IGF1 and IGF1R and protein expression of GFAP and vimentin) partially confirming our hypothesis; 4) The neonatal leptin treatment exerted a number of behavioral and neural effects that, in some cases, differently affected MD and control non MD animals (e.g., rearing frequency, expression of the following proteins: NeuN, PSD95, NCAM, CB1, NG2 and mRNA expression of IGF1 and IGF1R, CRH and LeptR); and 5) The vast majority of the effects caused by the treatments appeared to be sex and region-dependent.

Leptin signalling during the neonatal leptin surge, which peaks approximately at PND 9–10, has been shown to play a crucial role in the development of neuroendocrine circuits involved in metabolic control, particularly in the hypothalamus [50]. Leptin receptors are highly expressed in extra-hypothalamic brain regions and evidence is growing to indicate that leptin can regulate hippocampal synaptic function and influence many central processes including cognition. Moreover, several animal studies using adult leptin administration, as well as human studies, suggest that leptin is involved in the regulation of emotional behavior and neuropsychiatric disorders [34, 51]. In spite of these findings, there is scarce information about the programming effects of the neonatal leptin surge on the development of hippocampus and frontal cortex that are highly relevant for emotional control and cognitive function.

During the last years we have shown that rats deprived from their mothers during 24 h at PND 9 (coincident with the physiological leptin surge), MD animals, show a marked reduction in their leptin levels not only during the period of maternal separation but also afterwards, until PND 13 and even in the adulthood [26, 27]. These MD rats also show alterations in their developing hippocampus and frontal cortex, as well as behavioural modifications affecting their emotional status and cognitive function [16]. We hypothesised that the lack of appropriate neonatal leptin signalling in these MD animals could contribute to their neurodevelopmental alterations. To test this hypothesis, we have treated male and female animals with leptin during the critical days for leptin’s organizational effects (PND 9–13) and analysed their behaviour, hippocampus and cerebral cortex in the adulthood.

### Behavioral tests

In the open field, MD caused an increase in the percentage of internal ambulation in females which indicates a decreased “fear” for the open spaces (an anxiolytic-like effect). In the elevated plus maze, these same animals (MD females) showed an abnormally increased falls from the maze, and this occurred because these animals displayed a marked risk-taking behavior (such as rearing in open arms and peer into the void with more than half of the body outside the maze). It is known that leptin plays a role in depression- and anxiety- related behaviors. Indeed, administration of leptin (1 mg/kg ip) in adult rats [52] and mice [53] produces an antidepressant effect. In addition, there is evidence that leptin has anxiolytic-like properties, which can be observed in social interactions of mice after acute leptin administration [53]. The results reported here indicate that early modification of leptin levels might have long term emotional effects, which emphasizes the neuroprogramming effect of neonatal leptin signaling outside of the hypothalamus. In the open field test, leptin treatment increased the total ambulation and rearing frequency only in MD rats; thus, in this case MD appears to be a factor of vulnerability for the effect of leptin. There was a clear sexual dimorphism in these behavioral responses, since females showed more motor and exploratory activity than males, a finding that is in agreement with most of the literature (46–48).

Regarding cognition, no effect of MD was found in the novel object test. However, we have previously reported that both, adolescent MD (14) and adult MD animals [12] have a decreased discrimination index in this test, i.e., an impaired memory function. It is important to point out that the animals used in this study were injected several days during the neonatal critical period and this manipulation may have somehow protected the MD animals from the above mentioned changes. For example, neonatal handling, could have induced some resilience to the negative impact the MD protocols by increasing the quality and quantity of maternal care once the pups are returned to their mothers after the injection (handling) protocol [54]. In previous studies [18], we found that licking-grooming frequency was increased by MD during PND10 (after returning the pups to the dam). This is important because it has been proposed that an increase in maternal care may buffer or compensate the negative consequences of long mother/pup separations [55]. However, we have seen in many previous studies that the effects of this particular MD protocol are present [40], therefore, it is very likely that, in the present study, the absence of some of the effects of MD is rather attributable to the stress of injections. In this test a sexual dimorphism was found with females showing a greater discrimination index than males.

### Protein levels of synaptic plasticity markers

MD has been reported to notably affect several molecules implicated in synapses formation and stabilization [16]. In this study we found two significant interactions, i. e., sex x MD and sex x leptin, affecting hippocampal NeuN (MD increased the expression of this protein in males) and hippocampal synaptophysin, with MD males showing lower levels and MD females showing higher levels than their respective control groups. In a previous study on adolescent rats of both sexes, we found that MD decreased the expression of plasticity markers in the hippocampus and frontal cortex, including NeuN, BDNF, PSD95, synaptophysin and NCAM levels, and caused impaired recognition memory in the novel object test (NOT) [15]. We also previously found that adult MD animals showed impaired memory and changes in markers of synaptic plasticity in the hippocampus (mRNA levels of BDNF and synaptophysin) [12]. Moreover, a decrease in BDNF (mRNA and protein levels) was described in the hippocampal formation of adult male MD animals, although BDNF abnormalities exclusively appeared after weaning (PND21) [56]. Thus, we believe that, as previously indicated, the manipulation (daily injections from PND9 to PND 13) may have masked some of the potential MD effects, for example the lack of effect on BDNF in the present MD rats may be related with the lack of effect on their memory function. This interpretation is supported by the lack of effects of MD on leptin levels since in previous studies where early neonatal injections were not performed, we observed that MD diminished leptin levels in adulthood [12, 25]. However, as in previous studies, we did find, as expected, a sexual dimorphism [12, 25], with males having higher leptin levels than females.

Interestingly, we also observed several sex dependent effects of the neonatal leptin treatment on the above mentioned neural and synaptic plasticity markers. In particular leptin treated females showed an increase of NeuN expression in their hippocampus. This result is in agreement with previous in vivo and in vitro studies showing that leptin stimulates adult neurogenesis through direct receptor activation on newly formed neurons in the hippocampus and hypothalamus [57] and that leptin deficiency (ob/ob or db/db models) provokes neurodegeneration in frontal cortex and hippocampus. Leptin treated females also showed a significant decrease in hippocampal CB1 receptors expression. General effects of leptin consisting in a reduction of hippocampal synaptophysin and NCAM (140 KDa) levels, were also found, which together suggests decreased synapses possibly due to impaired synaptogenesis. On the other hand, in the cortex leptin increased PSD95 in females and NCAM and CB1 in both sexes. It is likely that, at least in relation to some of the parameters analyzed, the dose of leptin used has resulted in an excess of leptin during the neonatal period leading to disruption of synaptogenesis which manifests as an altered profile of synaptic plasticity markers in adulthood. The nature of these changes is mainly sex dependent, possibly reflecting differential temporal and/or functional developmental profiles in males and females. In general, leptin tended to decrease the parameters analyzed in hippocampus and increase them in the frontal cortex.

Given the increasing interest in the study of endocannabinoid system, it is worth emphasizing the importance of the CB1 cannabinoid receptor in the context of this study. The CB1 receptor is crucial for brain development and synaptic plasticity, as well as for the regulation and response to stress. We have repeatedly found clear short and long-term effects of MD on several components of the endocannabinoid system, including the CB1 receptor [16]. Under the present conditions, we observed modest effects of MD on hippocampal CB1 protein expression, but the neonatal leptin treatment significantly decreased CB1 expression in the hippocampus of females and increased cortical CB1 expression in both sexes. The relationship between leptin and the endocannabinoid system has been mainly analyzed in the context of energy homeostasis and in the hypothalamus [58]. Less is known regarding cannabinoid-leptin interactions in extra-hypothalamic areas, but given the pleiotrophic nature of both systems, it is very likely that they are involved in functions beyond energy homeostasis. Our results indicate that a deregulation of the postnatal leptin surge, due to a deficiency or an excess, has a long-term effect on the expression of CB1 receptor.

### mRNA expression of gens related with leptin and stress

The IGF system plays a central role in brain development and plasticity [59, 60] and one of its effects in the brain is to protect neurons from apoptosis [60]. In fact, we have shown in previous studies how MD modulates mRNA levels of IGF1 and its receptor in the hypothalamus [26]. For this reason, we studied this parameter here. Moreover, leptin modulates cell proliferation and differentiation [61], so we expected to find an effect of the neonatal leptin treatment. The expression of mRNA for IGF1 and its receptor increased in the hippocampus of MDVh males and neonatal leptin reversed these effects, whereas no effects of MD were found in Vh females in this area. In contrast, in the frontal cortex, MDVh males had a decreased expression of cortical IGF1 and IGF1R and these effects were not reversed by leptin. Thus, both, the effect of MD and the ability of leptin to reverse the effect of this neonatal stress is sex dependent.

Leptin tended to decrease the expression of mRNA for IGF1 and its receptor, with the exception of the frontal cortex in females. IGF-1 has been described as a potential biomarker for mood disorders [62] and, as indicated above, leptin has also been implicated in anxiety and depression related responses [34]; thus, it is possible that leptin and IGF-1 interact in the regulation of central nervous system functions.

One of the striking characteristics of prolonged maternal separation is the profound activation of the HPA. Down-regulation of CRH mRNA has been observed in mice exposed during 8 h to maternal separation, an effect which suggests direct modulation of the neonatal HPA axis [63]. Here we found that MD induced a long-term decrease in CRH mRNA levels in the frontal cortex of vehicle treated males and females. We found that this MD protocol markedly increases the levels of corticosterone (CORT) and this is evident at both PND10 and PND 13 [64]. It is plausible that this marked surge of CORT in this critical neonatal period programs HPA functioning. However, we cannot rule out that the neonatal Vh injections have changed the corticosterone response in MD rats. The neonatal leptin treatment induced a long-term decrease in hippocampal CRH mRNA levels in control non MD animals of both sexes and in MD males. Leptin feeds back to the hypothalamus to inhibit CRH release [34], so it is possible that it acts in a similar way in other brain regions. In the hippocampus of females the combination of MD and leptin treatment tended to normalize CRH mRNA levels, suggesting a complex interaction between the two treatments.

Finally, neonatal leptin treatment induced a long term effect on leptin receptor mRNA expression increasing this parameter in males and females (with the exception of control Vh males).

### Protein levels of glial markers

Leptin plays an important role in glial development [38, 39, 65] and we have previously found that MD also affects glial cells development [16]. Under the present experimental conditions, the expression of the astrocyte marker GFAP decreased in both brain regions in MD males and in the frontal cortex of females. Leptin treatment reversed the MD effects in males, but not in females. The other astrocyte marker analyzed, vimentin, was also decreased in the hippocampus and frontal cortex of MD females, and in these cases (especially in the hippocampus) neonatal leptin treatment reversed this effect. As early stress induces cell death [66], in view of the decrease in glial markers, it is plausible that MD might have induced glial death. On the other hand, leptin is implicated in brain progenitor cell proliferation and astrocyte differentiation in the hypothalamus [67]. Moreover, astrocytic and neuronal leptin signaling interacts with each other in the execution of normal and pathophysiological functions in extrahypothalamic brain areas such as the hippocampus and frontal cortex [68]. The present data point to an efficient protective effect of the leptin treatment in MD animals and suggest a clear interaction between both MD and neonatal leptin exposure. The potential clinical utility of leptin through its modulation of neuron-glia interaction remains to be determined.

MD females also showed decreased expression of the oligodendrocyte precursor NG2 in their frontal cortex, with this effect being partially counteracted by neonatal leptin. In line with this finding, an increase in the rate of cell death, mostly of oligodendrocytes in white matter tracts, was observed by Zhang *et al*. (2002) following a similar protocol of early life stress (MD at PND 11). There was also a clear sex difference in the frontal cortex between the CoVh groups, with females showing higher levels of NG2.

GLAST expression is altered in pathological conditions, such as hypoxia/ischemia, multiple sclerosis, schizophrenia and epilepsy. In general, activity of this glutamate transporter is considered to be responsible for termination of glutamatergic transmission and for the prevention of excitotoxic damage [69]. Stress leads to glutamate release in the hippocampus [70], and over-activation can put the neuron at risk of excitotoxicity [71, 72]. Pickering *et al*. (2006) found an increase in GLAST expression in the hippocampus of MD males (360 min of daily maternal deprivation during PND1-21). Here we show an increase in GLAST protein expression in the frontal cortex of MD males that tended to be compensated by the neonatal leptin treatment, whereas the opposite was found in females, i.e., a modest decrease in GLAST frontal cortex expression in all MD females. Since GLAST is found mainly in astrocytes and other glial cells [73, 74], this decrease of GLAST expression in MD females might be related to their diminished expression of the markers of glial cells.

## Conclusions

In summary, in some but not all of the parameters analyzed, the neonatal leptin treatment reversed the effects of MD, partially confirming our hypothesis. In those cases where the leptin treatment did not reverse the effects of MD, the reason might be that MD is a complex neonatal stress involving not only a marked decrease of leptin levels, but also an increase in corticosterone and a decrease in glucose levels [40]. In fact, MD involves lack of social contact with the mother, lack of milk ingestion, dehydration and hypothermia, all of which could be involved in the long-term consequences.

We found that some of the previously observed effects of this neonatal stress were not found this study. We propose that the stress of the injections administered during the critical neonatal period may have exerted a protective effect (or could have changed the “baseline” in the controls). This hypothesis opens new questions regarding the interaction of different manipulations or events during early life.

The neonatal leptin treatment, per se, exerted a number of effects on synaptic plasticity markers. The data support the hypothesis that the neonatal leptin surge plays an important role in the development of extrahypotalamic areas and that an adequate leptin level, avoiding both excess and deficiency, appears to be necessary for its correct neuroprograming effect. These concepts may have clinical implications and deserve to be studied further in the future [34]. A potential clinical application of leptin should evaluate the optimal level required depending on the age and sex of the individual and should also consider that the effect of leptin may be region dependent.

The data presented revealed diverse sexual dimorphisms that are probably due to the organizational effects of perinatal gonadal hormones during a critical period of brain sexual differentiation [75, 76], and/or to their organizational effects and the activation they produce during the peri-adolescent period [77, 78]. Another complementary factor that may contribute to the sex differences observed is the different neurodevelopmental stage of males and females at the ages when the treatments are performed [79].
